# Blunt versus penetrating trauma: differences in early physiology, coagulopathy, and outcomes in a Scandinavian trauma centre

**DOI:** 10.1186/s13049-026-01683-5

**Published:** 2026-08-29

**Authors:** Erik von Oelreich, Jesper Eriksson, Bjarni Arnason, Daniel Ohlén, Axel Hedin, Anders Gille, Malin Jonsson Fagerlund, Magnus Hedberg

**Affiliations:** 1 Perioperative Medicine and Intensive Care, Karolinska University Hospital, Solna, Stockholm, SE-171 76 Sweden; 2 Section for Anesthesiology and Intensive Care Medicine, Department of Physiology and Pharmacology, Karolinska Institutet, Stockholm, Sweden

**Keywords:** Trauma, Penetrating trauma, Blunt trauma, Resuscitation, Transfusion, Coagulation, Outcome

## Abstract

**Background:**

Blunt and penetrating trauma differ in injury patterns, management, and outcomes, but data comparing early physiological and haemostatic responses within Scandinavian trauma systems remain limited. We aimed to investigate differences in clinical characteristics, early physiology, and outcomes between blunt and penetrating trauma patients at a major Swedish trauma centre.

**Methods:**

This registry-based observational cohort study included adult trauma patients admitted to the Karolinska University Hospital, Stockholm, between November 2022 and December 2024. Detailed prospective data were collected for patients receiving blood transfusion within two hours of arrival. Patients were categorised by injury type (blunt vs. penetrating). Clinical outcomes included 30-day mortality, hospital length of stay, and functional outcome. Secondary analyses compared physiological, metabolic, and coagulation parameters. Multivariable logistic regression was used to compare 30-day mortality between groups, adjusted for age, sex, Injury Severity Score, and preinjury comorbidity.

**Results:**

A total of 2,193 patients were included, of whom 353 (16%) sustained penetrating trauma. Patients with penetrating trauma were younger (median 34 vs. 49 years, *p* < 0.001), more often male (78% vs. 66%, *p* < 0.001), and more frequently presented in shock (5.9% vs. 2.2%, *p* < 0.001), with a greater need for emergency surgery (53% vs. 30%, *p* < 0.001) and early blood transfusion (20% vs. 4.5%, *p* < 0.001). Blunt trauma patients had higher injury severity (median ISS 10 vs. 4, *p* < 0.001), longer hospital stays (> 7 days: 26% vs. 18%, *p* = 0.001), and worse functional outcomes at discharge (Glasgow Outcome Scale 2 or 3: 40% vs. 12%, *p* < 0.001). In the early transfusion subgroup (n = 152), blunt trauma patients exhibited lower fibrinogen levels (median 2.1 vs. 2.5 g/L, p = 0.027), lower platelet counts (median 239 vs. 271 10⁹/L, p = 0.011), and prolonged activated partial thromboplastin time (median 26 vs. 23 s, p < 0.001) compared with penetrating trauma patients, despite similar rates of trauma-induced coagulopathy defined by INR > 1.2 (23% vs. 16%, p = 0.32). After exclusion of non-trauma-related deaths, 30-day mortality did not differ significantly between groups in unadjusted or adjusted analyses.

**Conclusions:**

Within this Scandinavian trauma system, blunt and penetrating trauma represent distinct clinical entities with differing injury patterns, physiological responses, and management needs. Despite these differences, adjusted mortality was comparable. These findings may support mechanism-specific assessment and prioritisation in trauma management.

**Trial registration:**

This study is a secondary analysis of a prospectively registered observational trauma cohort (ClinicalTrials.gov NCT05573841, registered 6 October, 2022).

## Background

Trauma is a major contributor to mortality and morbidity across all age groups, with a particularly high burden among young adults [1]. Globally, the majority of trauma patients sustain injuries from blunt mechanisms; however, the proportion of blunt versus penetrating trauma varies considerably between geographical regions [2]. In recent years, the management of penetrating trauma has received increased attention, partly driven by ongoing armed conflicts worldwide. In Sweden, there has been a marked rise in penetrating trauma over the past decade, largely attributable to gang-related gun violence [3–5]. The Swedish national trauma registry has in recent years reported that penetrating injuries account for more than 8% of all trauma cases, with the exception of a downward trend observed in 2024 [6]. Despite the increase in gun-related violence, stab wounds remain the predominant mechanism of penetrating trauma in Sweden [3, 4, 6].

Patients sustaining penetrating trauma are typically young males with injuries confined to one or two anatomical regions, where uncontrolled haemorrhage constitutes the primary immediate threat to life [3, 7, 8]. In contrast, blunt trauma patients exhibit a broader age distribution and frequently sustain multisystem injuries, often including traumatic brain injury (TBI) [9].

Although differences in injury severity, management, and outcomes between blunt and penetrating trauma have been reported, several previous studies originate from trauma systems with substantially different epidemiology from Scandinavia, particularly regarding the prevalence of firearm-related injuries [10–12]. Data comparing early physiological, metabolic, and coagulation disturbances within Scandinavian trauma systems remain limited.

Improved understanding of how injury mechanism influences the early physiological response to trauma may inform clinical decision-making and resuscitation strategies. We therefore hypothesised that patients with penetrating trauma present with more pronounced physiological and metabolic derangement on arrival to hospital but, due to more isolated injury patterns and greater physiological reserve, have more favourable outcomes than patients sustaining blunt trauma.

The aim of this study was to characterise differences in injury patterns, early physiological and haemostatic responses, management, and clinical outcomes between blunt and penetrating trauma patients admitted to a major Swedish trauma centre. Clinical outcomes included 30-day mortality, hospital length of stay, and functional outcome at discharge. A predefined subgroup of patients requiring blood transfusion in the prehospital phase or within two hours of hospital arrival underwent more detailed analyses of physiological, metabolic, and coagulation parameters.

## Methods

### Ethics

The study was conducted in accordance with the Declaration of Helsinki. Ethical approval was granted by the Swedish Ethical Review Authority (Dnr 2021–04394), with a waiver of informed consent. The study is reported in accordance with the STROBE statement for observational studies (checklist provided as Additional Material).

### Study design and setting

This registry-based observational cohort study used data from the Karolinska Trauma Registry for patients admitted between November 1, 2022, and December 31, 2024. For patients receiving blood transfusion in the prehospital phase or within two hours of hospital arrival, additional prospectively collected study-specific laboratory and transfusion data were available. The present study was a secondary analysis of a prospectively registered observational trauma cohort (ClinicalTrials.gov identifier: NCT05573841) and was not designed or powered primarily to detect differences in mortality between injury mechanisms.

Karolinska University Hospital is the primary and only regional trauma centre in the Stockholm region, serving a predominantly urban catchment area of approximately 2.5 million inhabitants. In the Stockholm region, all patients meeting the Swedish national level I trauma team activation criteria are transported directly to the Karolinska University Hospital. This includes all patients with penetrating injuries proximal to the elbow or knee. Trauma patients bypass the emergency department and are admitted directly to the trauma unit, which has immediate access to computed tomography, operating theatres, and interventional radiology.

Patients with suspected major haemorrhage were managed according to local trauma and massive transfusion protocols. Administration of blood products was based on clinical judgement incorporating evidence of active bleeding, haemodynamic status, viscoelastic testing (ROTEM), and conventional coagulation tests.

### Prehospital care

The regional prehospital emergency medical services consist primarily of ground ambulance units, supplemented by helicopter emergency medical services for patients located in the Stockholm archipelago or when transport times can be reduced. Physician-staffed rapid response units support the prehospital system and may be dispatched to life-threatening emergencies, including major trauma. These units are equipped to provide advanced treatment interventions and may carry whole blood or blood components depending on unit configuration.

### Healthcare context and trauma registry

Sweden operates a tax-funded universal healthcare system. Each resident is assigned a unique personal identity number, enabling linkage across national healthcare registers with high completeness and accuracy [13]. Trauma data were obtained from the Swedish Trauma Registry, a national quality registry that prospectively records information on trauma patients admitted following trauma team activation.

The registry includes data on injury mechanism, physiological parameters, injury severity scoring, prehospital and in-hospital time intervals, and acute interventions. Patients with isolated limb injuries, drowning, hanging without traumatic injury, accidental hypothermia without concomitant trauma, chronic subdural hematoma, or burns involving less than 18% total body surface area without inhalation injury are not included in the registry. At the Karolinska University Hospital, registry nurses perform continuous data entry and regular manual reviews of admission records from relevant departments to identify eligible patients.

### Study population

All adult patients aged 15 years or older admitted via the trauma unit during the study period and recorded in the Karolinska Trauma Registry were eligible for inclusion. Patients declared dead on arrival without subsequent in-hospital interventions were not included in the study population. Patients were categorised according to injury mechanism as blunt or penetrating trauma. The early blood transfusion subgroup was defined as patients who received at least one unit of blood product (whole blood, packed red blood cells, plasma, or platelets) either in the prehospital phase or within 2 h of hospital arrival. Patients with a non-trauma-related cause of death within 30 days were excluded from mortality analyses only.

Patients in the early transfusion group were initially identified by anaesthetic nurses or physicians who were members of the trauma team, and a study-specific research form was completed at admission. All trauma admissions were subsequently reviewed by a study investigator to ensure completeness of inclusion.

### Data sources and variables

Data were collected from multiple sources, including the study-specific research form completed in the trauma unit, the Swedish Trauma Registry, electronic medical records, anaesthesia charts, and the blood bank laboratory database. Collected variables included patient demographics, injury characteristics, physiological parameters, blood gas and coagulation analyses, administration of procoagulant agents and calcium, transfusion type and volume, and outcome measures. Traumatic coagulopathy was defined as International Normalised Ratio (INR) > 1.2. Shock was defined as a systolic blood pressure < 90 mmHg.

### Statistical analysis

Logistic regression analyses were conducted to estimate the odds of death associated with penetrating versus blunt trauma. An unadjusted model was first fitted, followed by a model adjusted for age and sex, and a fully adjusted model including age, sex, Injury Severity Score (ISS), and preinjury American Society of Anesthesiologists (ASA) classification. No formal missing data analysis was performed. All analyses were conducted on patients with available data for each variable (complete case analysis). Data completeness was high throughout the study period, owing to prospective registry data collection, continuous data entry by registry nurses, and retrospective review of all trauma admissions by a study investigator. Survival over time was assessed using Kaplan–Meier analysis.

Categorical variables were reported as counts and percentages, and continuous variables as medians with interquartile ranges or means with standard deviations, as appropriate. Group comparisons were performed using Student’s t-test for normally distributed continuous variables, Mann–Whitney U test for non-normally distributed continuous variables, chi-square test for categorical variables, and Kruskal–Wallis test for comparisons involving ≥ 3 groups. A two-sided p-value < 0.05 was considered statistically significant. No adjustment for multiple comparisons was performed. Apart from the mortality analyses, comparisons of physiological, metabolic, haemostatic, and treatment variables should therefore be interpreted as exploratory. Statistical analyses were performed using Stata version 19 (StataCorp, College Station, TX, USA).

### Artificial Intelligence

The authors used ChatGPT (OpenAI) for language editing. The authors reviewed and verified all generated content and take full responsibility for the manuscript.

## Results

### Study population

**Fig. 1:**
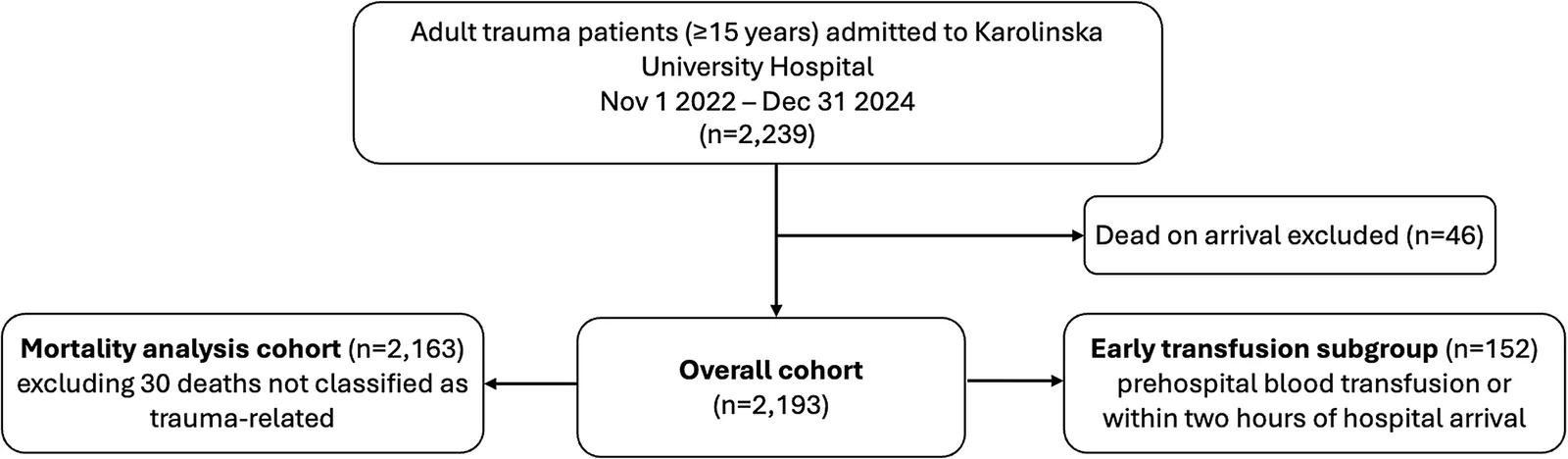
Flow chart of included patients

The overall cohort comprised 2,193 patients, of whom 353 (16%) sustained penetrating trauma and 1,840 (84%) blunt trauma (Fig. 1). After exclusion of 29 patients with a non-trauma-related cause of death and one patient in whom the relation to trauma could not be determined, 2,163 patients remained in the mortality analysis cohort, which formed the basis for the logistic regression analyses. The early blood transfusion subgroup consisted of 152 patients, including 70 (46%) with penetrating trauma and 82 (54%) with blunt trauma. Baseline characteristics and trauma room characteristics are presented in Tables 1 and 2.

**Table 1:** Prehospital and baseline characteristics stratified by penetrating versus blunt trauma

Factor	Penetrating	Blunt	*p*-value
**N**	353	1840	
Age, median (IQR)	34 (24, 46)	49 (31, 66)	< 0.001
Male sex, n (%)	276 (78%)	1208 (66%)	< 0.001
**ASA preinjury**,** n (%)**			0.019
1	157 (45%)	715 (39%)	
2	129 (37%)	643 (35%)	
3	65 (18%)	448 (24%)	
4	2 (1%)	34 (2%)	
**Injury mechanism**,** n (%)**			< 0.001
Traffic	1 (0.3%)	713 (39%)	
Shot by gun	75 (21%)	0 (0%)	
Stabbed by knife	275 (78%)	0 (0%)	
Struck by blunt object	0 (0%)	178 (10%)	
Fall	0 (0%)	862 (47%)	
Other	2 (1%)	85 (5%)	
**First recorded GCS**,** n (%)**			< 0.001
13–15	295 (92%)	1124 (78%)	
9–12	7 (2%)	112 (8%)	
3–8	18 (6%)	214 (15%)	
**Highest level of prehospital care**,** n (%)**			< 0.001
No Field Care	14 (4%)	51 (4%)	
ALS paramedic - no physician	166 (50%)	1142 (76%)	
ALS paramedic and physician	155 (46%)	316 (21%)	
**Prehospital cardiac arrest**,** n (%)**	11 (3%)	29 (2%)	0.12
**Time on scene (min)**,** median (IQR)**	15 (10, 21)	22 (15, 29)	< 0.001
**Transport time (min)**,** median (IQR)**	16 (11, 21)	15 (11, 21)	0.61

Categorical parameters are presented as n (%), continuous parameters as median with interquartile range (IQR). ALS, advanced life support; GCS, Glasgow Coma Scale; IQR, interquartile range

**Table 2:** Trauma room characteristics and outcomes stratified by penetrating versus blunt trauma

Factor	Penetrating	Blunt	*p*-value
**N**	353	1840	
**ISS**,** median (IQR)**	4 (1,10)	10 (4,18)	< 0.001
**NISS**,** median (IQR)**	5 (2,16)	12 (4,26)	< 0.001
**Shock on arrival**,** n (%)**	21 (6%)	41 (2%)	< 0.001
**First Base Excess**,** median (IQR)**	0.0 (-3.0, 1.9)	0.6 (-2.0, 2.0)	0.030
**Intubation**,** n (%)**			0.33
Intubated at trauma bay	32 (10%)	150 (10%)	
Not intubated	278 (86%)	1227 (83%)	
Intubated prehospital	15 (4%)	99 (7%)	
**First emergency procedure**,** n (%)**			< 0.001
DC thoracotomy	10 (3%)	8 (0%)	
DC laparotomy	41 (12%)	33 (2%)	
Limb revascularisation	7 (2%)	4 (0%)	
Interventional radiology	5 (1%)	22 (1%)	
Craniotomy	1 (0%)	84 (5%)	
ICP device insertion	3 (1%)	30 (2%)	
Other	121 (34%)	374 (20%)	
No emergency intervention	165 (47%)	1282 (70%)	
**Time to CT**,** n (%)**			< 0.001
Time to CT ≤ 30 min	202 (69%)	898 (55%)	
Time to CT 31–60 min	42 (14%)	377 (23%)	
Time to CT > 60 min	47 (16%)	350 (22%)	
**Time to first emergency procedure**,** n (%)**			< 0.001
≤30 min	42 (22%)	56 (10%)	
31–60 min	45 (24%)	62 (11%)	
>60 min	103 (54%)	447 (79%)	
**Highest level of care**,** n (%)**			< 0.001
Emergency Department	48 (14%)	228 (12%)	
General Ward	118 (33%)	706 (38%)	
Operation Theatre	124 (35%)	349 (19%)	
HDU	13 (4%)	145 (8%)	
Intensive Care Unit	50 (14%)	412 (22%)	
**Hospital LOS**,** n (%)**			< 0.001
≤2 days	202 (57%)	712 (39%)	
3–7 days	88 (25%)	649 (35%)	
>7 days	63 (18%)	479 (26%)	
**GOS at discharge**,** n (%)**			< 0.001
Death	13 (4%)	99 (5%)	
Persistent vegetative state	0 (0%)	5 (0%)	
Severe disability	43 (12%)	728 (40%)	
Moderate disability	105 (30%)	475 (26%)	
Good recovery	192 (54%)	533 (29%)	
**30-day mortality**,** n (%)**	14 (4%)	134 (7%)	0.022
**Trauma-related death**,** n (%)**			0.027
Not trauma-related	0 (0%)	29 (22%)	
Trauma-related	13 (93%)	104 (78%)	
Unknown	1 (7%)	1 (1%)	
**Cause of death**,** n (%)**			< 0.001
TBI	3 (21%)	65 (49%)	
Haemorrhagic shock	8 (57%)	3 (2%)	
MOF	0 (0%)	7 (5%)	
Other	3 (21%)	59 (44%)	

Categorical parameters are presented as n (%), continuous parameters as median with interquartile range (IQR). ISS, Injury Severity Score; NISS, New Injury Severity Score; CT, computed tomography; DC, damage control; ED, emergency department; GOS, Glasgow Outcome Scale; HDU, High Dependency Unit; ICP, intracranial pressure; LOS, length of stay; MOF, multiple organ failure; TBI, traumatic brain injury

### Mortality analyses

**Fig. 2:**
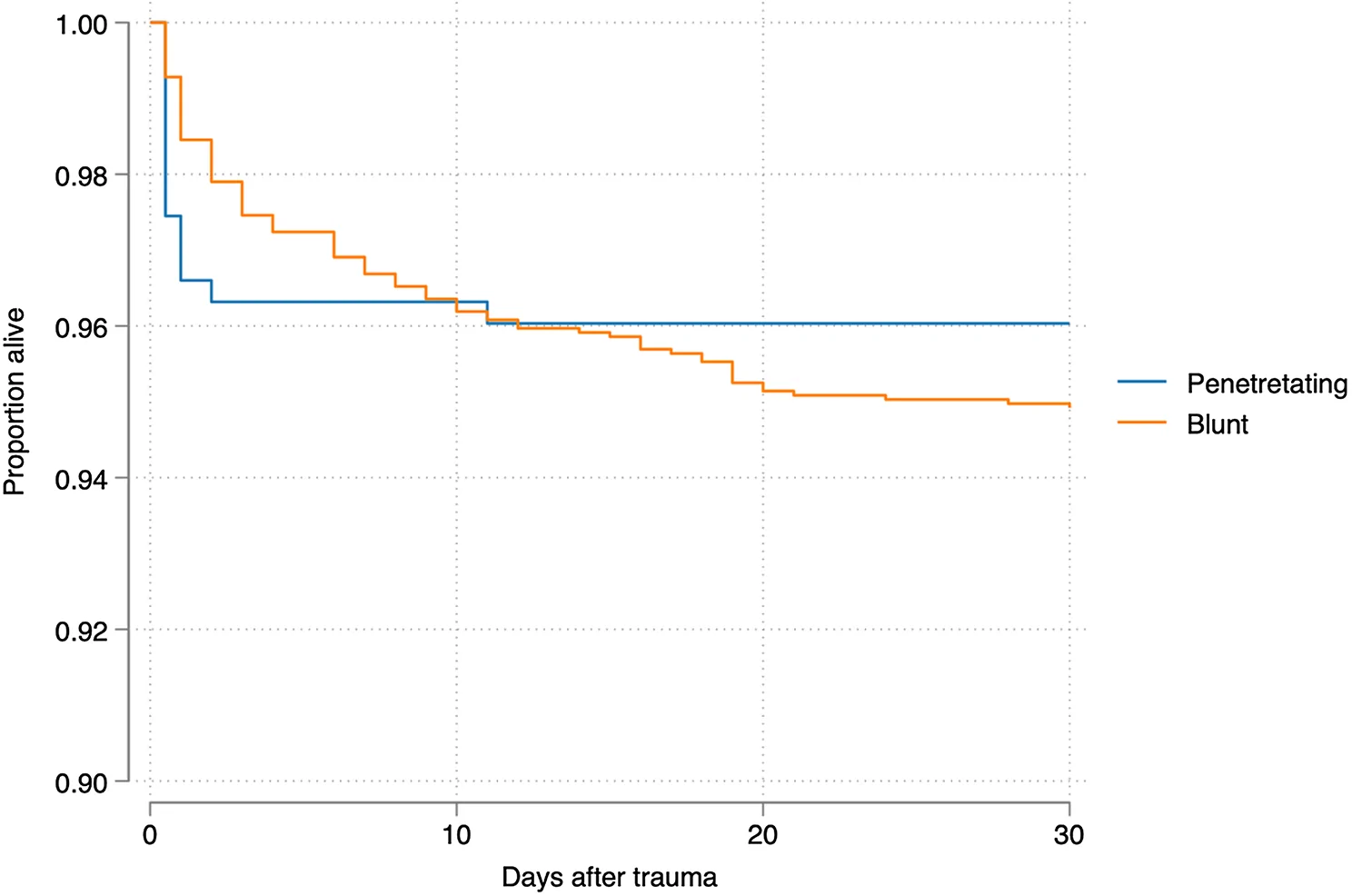
Kaplan–Meier survival curves stratified by injury type. Kaplan–Meier estimates of survival up to 30 days after trauma comparing patients with penetrating versus blunt injury. The x-axis shows days after trauma and the y-axis shows proportion alive

Kaplan–Meier survival curves comparing blunt and penetrating trauma are shown in Fig. 2. Mortality following penetrating trauma occurred predominantly during the first day after admission, with only one death occurring beyond day 3. In contrast, mortality after blunt trauma was distributed throughout the 30-day follow-up period.

No statistically significant difference in 30-day mortality between penetrating and blunt trauma was observed in any of the regression models. The odds ratio for penetrating versus blunt trauma was 0.63 (95% CI 0.35–1.13; *p* = 0.12) in the unadjusted analysis, 1.03 (95% CI 0.56–1.90; *p* = 0.93) after adjustment for age and sex, and 1.46 (95% CI 0.60–3.55; *p* = 0.40) in the fully adjusted model including age, sex, ISS, and ASA classification. The unadjusted and age- and sex-adjusted models included 2,148 patients, and the fully adjusted model 2,147 patients.

Causes of death are presented in Table 2. In the blunt trauma group, the cause of death was TBI in 49% and haemorrhage in 2.2%. In the penetrating group, the proportion of deaths from haemorrhage was 57% and 21% from TBI.

### Prehospital and baseline characteristics

Prehospital and baseline characteristics stratified by injury type are presented in Table 1. Compared with blunt trauma, patients with penetrating trauma were younger, more often male, and had lower preinjury ASA classifications. Blunt mechanisms were dominated by falls and traffic-related injuries, while penetrating trauma was most commonly caused by stab wounds.

### Trauma room characteristics and in-hospital outcomes

Trauma room characteristics and in-hospital outcomes are presented in Table 2. Patients with penetrating trauma more frequently presented with shock on arrival and more often underwent emergency surgery, particularly damage control laparotomy and thoracotomy. In contrast, patients with blunt trauma more frequently had higher injury severity, greater need for intensive care, longer hospital stay, and poorer functional outcome at discharge.

### High-resolution prehospital and in-hospital parameters

High-resolution prehospital and early in-hospital parameters for patients receiving early blood transfusion are presented in Table 3. Admission lactate, base excess, pH, haemoglobin, and ionised calcium were similar between groups.

Despite similar INR-defined traumatic coagulopathy and largely similar viscoelastic findings, blunt trauma patients had lower fibrinogen levels, lower platelet counts, and prolonged activated partial thromboplastin time.

**Table 3:** High-resolution prehospital and in-hospital parameters in patients receiving early blood transfusion

Factor	Penetrating	Blunt	*p*-value
**N**	70	82	
**Prehospital administration and transfusion**			
**Tranexamic acid**,** n (%)**	41 (59%)	44 (54%)	0.54
**Crystalloid (mL)**,** mean (SD)**	99 (222)	205 (403)	0.053
**Whole blood transfusion**,** n (%)**	19 (27%)	17 (21%)	0.35
**PRBC transfusion**,** n (%)**	12 (17%)	6 (7%)	0.062
**Plasma transfusion**,** n (%)**	9 (13%)	4 (5%)	0.080
**Blood transfusion of any product**,** n (%)**	31 (44%)	24 (29%)	0.055
**Laboratory testing at arrival to the trauma unit**			
**Lactate**,** median (IQR)**	4.8 (3.0, 8.7)	4 (2.7, 8.4)	0.31
**Base Excess**,** median (IQR)**	-3.2 (-8.5, -0.5)	-4.7 (-12.0, -2.0)	0.18
**pH**,** median (IQR)**	7.29 (7.14, 7.36)	7.26 (7.12, 7.32)	0.22
**Hb**,** median (IQR)**	126 (115, 147)	128 (115, 142)	0.95
**Ionized calcium (mmol/L)**,** median (IQR)**	1.15 (1.11, 1.20)	1.17 (1.13, 1.21)	0.31
**Fibrinogen**,** median (IQR)**	2.5 (2.0, 2.9)	2.1 (1.7, 2.6)	0.027
**APTT**,** median (IQR)**	23 (21, 26)	26 (23, 33)	< 0.001
**Platelet count**,** median (IQR)**	271 (208, 330)	239 (174, 286)	0.011
**INR**,** median (IQR)**	1.1 (1.0, 1.2)	1.1 (1.0, 1.2)	0.047
**EXTEM CT**,** median (IQR)**	65 (59, 75)	67 (60, 79.5)	0.37
**EXTEM A5**,** median (IQR)**	46 (39, 51)	43 (38, 49)	0.057
**EXTEM ML**,** median (IQR)**	6 (3, 10)	4 (2, 8)	0.005
**FIBTEM A5**,** median (IQR)**	11 (9, 14)	11 (7, 14)	0.46
**Trauma-induced coagulopathy**,** n (%)**	10 (16%)	17 (23%)	0.32
**Massive transfusion**,** n (%)**	37 (54%)	55 (67%)	0.092
**Tranexamic acid total dose**,** first 3 h**,** n (%)**			0.18
0 g	8 (12%)	14 (17%)	
1 g	21 (30%)	27 (33%)	
2 g	38 (55%)	36 (44%)	
>2 g	2 (2%)	4 (5%)	
**Transfusions during the first 24 h**			
**Whole blood (mL)**,** median (IQR)**	456 (0, 1346)	459 (0, 954)	0.88
**PRBC (mL)**,** median (IQR)**	531 (250, 1286)	1028 (272, 1999)	0.083
**Plasma (mL)**,** median (IQR)**	513 (245, 1774)	970 (252, 1802)	0.20
**Platelets (mL)**,** median (IQR)**	0 (0, 207)	196 (0, 395)	0.021
**Total transfusion (mL)**,** median (IQR)**	1702 (901, 4021)	2659 (1164, 5370)	0.12

Categorical parameters are presented as n (%), continuous parameters as median with interquartile range (IQR) or mean with standard deviation (SD). APTT, activated partial thromboplastin time; EXTEM/FIBTEM, rotational thromboelastometry parameters; Hb, haemoglobin; INR, International Normalized Ratio; PRBC, Packed Red Blood Cells. Early blood transfusion was defined as prehospital or within 2 h of hospital arrival

Characteristics and comparisons between patients with stab wounds and gunshot wounds are presented in Tables 4 and 5. Patients with gunshot wounds were younger, had fewer comorbidities, higher injury severity, and higher mortality.

**Table 4:** Prehospital and baseline characteristics stratified by gunshot wound versus stab wound

Factor	Gunshot wound	Stab wound	*p*-value
**N**	75	275	
Age, median (IQR)	25 (19, 31)	36 (26, 47)	< 0.001
Male sex, n (%)	72 (96%)	202 (74%)	< 0.001
**ASA preinjury**,** n (%)**			< 0.001
1	58 (77%)	99 (36%)	
2	14 (19%)	114 (42%)	
3	3 (4%)	60 (22%)	
4	0 (0%)	2 (1%)	
**First recorded GCS**,** n (%)**			< 0.001
13–15	50 (77%)	242 (96%)	
9–12	3 (5%)	4 (2%)	
3–8	12 (19%)	6 (2%)	
**Highest level of prehospital care**,** n (%)**			0.002
No Field Care	2 (3%)	12 (5%)	
ALS paramedic - no physician	21 (31%)	143 (54%)	
ALS paramedic and physician	44 (66%)	110 (42%)	
**Prehospital cardiac arrest**,** n (%)**	9 (14%)	2 (1%)	< 0.001
**Time on scene (min)**,** median (IQR)**	12 (9, 17)	16 (10, 22)	0.019
**Transport time (min)**,** median (IQR)**	13 (10, 19)	16 (12, 21)	0.016

Categorical parameters are presented as n (%), continuous parameters as median with interquartile range (IQR). ALS, advanced life support; GCS, Glasgow Coma Scale; IQR, interquartile range

**Table 5:** Trauma room characteristics and outcomes stratified by gunshot wound versus stab wound

Factor	Gunshot wound	Stab wound	*p*-value
**N**	75	275	
**Shock on arrival**,** n (%)**	11 (15%)	10 (4%)	< 0.001
**First Base Excess**,** median (IQR)**	-0.55 (-4.75, 1.0)	0.1 (-2.6, 2.0)	0.039
**ISS**,** median (IQR)**	14 (4, 29)	2 (1, 9)	< 0.001
**NISS**,** median (IQR)**	22 (4, 34)	3 (1, 9)	< 0.001
**Intubation**,** n (%)**			< 0.001
Intubated at trauma bay	14 (21%)	18 (7%)	
Not intubated	42 (62%)	233 (92%)	
Intubated prehospital	12 (18%)	3 (1%)	
**First emergency procedure**,** n (%)**			< 0.001
DC thoracotomy	8 (11%)	2 (1%)	
DC laparotomy	11 (15%)	30 (11%)	
Limb revascularisation	3 (4%)	4 (2%)	
Interventional radiology	0 (0%)	5 (2%)	
Craniotomy	1 (1%)	0 (0%)	
ICP device insertion	3 (4%)	0 (0%)	
Other	20 (27%)	99 (36%)	
No emergency intervention	29 (39%)	135 (49%)	
**Time to CT**,** n (%)**			0.16
Time to CT ≤ 30 min	42 (63%)	157 (71%)	
Time to CT 31–60 min	9 (13%)	33 (15%)	
Time to CT > 60 min	16 (24%)	31 (14%)	
**Time to first emergency procedure**,** n (%)**			0.009
≤30 min	18 (38%)	24 (17%)	
31–60 min	8 (17%)	37 (26%)	
>60 min	21 (45%)	80 (57%)	
**Highest level of care**,** n (%)**			< 0.001
Emergency Department	5 (7%)	43 (16%)	
General Ward	17 (23%)	100 (36%)	
Operation Theatre	30 (40%)	93 (34%)	
HDU	0 (0%)	13 (5%)	
Intensive Care Unit	23 (31%)	26 (10%)	
**Hospital LOS**,** n (%)**			< 0.001
≤2 days	31 (41%)	170 (62%)	
3–7 days	16 (21%)	71 (26%)	
>7 days	28 (37%)	34 (12%)	
**GOS at discharge**,** n (%)**			< 0.001
Death	11 (15%)	2 (1%)	
Severe disability	21 (28%)	22 (8%)	
Moderate disability	25 (33%)	78 (28%)	
Good recovery	18 (24%)	173 (63%)	
**30-day mortality**,** n (%)**	11 (15%)	3 (1%)	< 0.001
**Trauma-related death**,** n (%)**			0.59
Trauma-related	10 (91%)	3 (100%)	
Unknown	1 (9%)	0 (0%)	
**Cause of death**,** n (%)**			0.57
TBI	3 (27%)	0 (0%)	
Haemorrhagic shock	6 (55%)	2 (67%)	
Other	2 (18%)	1 (33%)	

Categorical parameters are presented as n (%), continuous parameters as median with interquartile range (IQR). CT, computed tomography; DC, damage control; ED, emergency department; GOS, Glasgow Outcome Scale; HDU, High Dependency Unit; ICP, intracranial pressure; ISS, Injury Severity Score; LOS, length of stay; MOF, multiple organ failure; NISS, New Injury Severity Score; TBI, traumatic brain injury

## Discussion

This study demonstrates that blunt and penetrating trauma represent distinct clinical entities within a Scandinavian trauma system. While penetrating trauma was characterised by greater early haemodynamic instability and high need for immediate surgical interventions, blunt trauma was associated with higher injury severity, greater need for intensive care, and poorer functional outcomes. Despite these differences in presentation and management, adjusted 30-day mortality did not differ significantly between the injury mechanisms. These findings suggest that injury mechanism influences the early clinical phenotype and management priorities.

The present findings should be interpreted in the context of Scandinavian trauma epidemiology. Penetrating trauma accounted for only 16% of admissions and was predominantly caused by stab wounds, despite the recent increase in firearm-related violence in Sweden [3, 4, 6]. This differs from trauma populations with a substantially higher proportion of firearm injuries [10], which may partly explain differences in injury severity and mortality between studies [10–12].

The distribution of causes of death further illustrates the different injury patterns associated with blunt and penetrating trauma. Among penetrating trauma patients, non-survivors typically died early from haemorrhagic shock, whereas traumatic brain injury was the predominant cause of death following blunt trauma. The predominance of TBI as the leading cause of death in blunt trauma in our cohort is consistent with prior studies from high-income trauma systems, where improvements in haemorrhage control have shifted the burden of mortality towards neurological injury [14]. Moreover, the number of deaths classified as ‘Other’ was high among blunt trauma patients, partly explained by the high number of non-traumatic deaths in this group.

Despite clear differences in injury patterns and early management, we did not observe a difference in adjusted 30-day mortality between penetrating and blunt trauma after excluding non-traumatic deaths. This finding should be interpreted cautiously, as the number of trauma-related deaths following penetrating trauma was small, resulting in limited statistical precision and wide confidence intervals. As firearm injuries are typically associated with more extensive tissue destruction and higher mortality [10], this may partly explain both the relatively low mortality observed and why our findings contrast with previous studies from settings with a higher prevalence of firearm-related injuries [11, 12].

Functional outcomes were also worse among blunt trauma patients at discharge, which is likely driven by the higher incidence of TBI, a well-established determinant of long-term disability after trauma [14]. This finding highlights that mortality alone does not fully capture the burden of blunt trauma, where long-term disability may represent an equally important outcome.

Among patients receiving early blood transfusion, differences in early haemostatic profiles were observed between blunt and penetrating injuries. Although the incidence of traumatic coagulopathy defined by INR did not differ and most viscoelastic parameters were similar between groups, blunt trauma patients demonstrated lower fibrinogen levels, lower platelet counts, and prolonged activated partial thromboplastin time compared with penetrating trauma patients. These findings suggest modest but distinct early haemostatic alterations following blunt trauma, possibly reflecting the combined effects of tissue injury, hypoperfusion, and inflammation [15, 16]. In contrast, penetrating trauma may more often represent a “pure” haemorrhagic phenotype, where bleeding is driven primarily by mechanical vascular disruption rather than systemic coagulopathy. These findings may have potential implications for resuscitation strategies and support careful attention to fibrinogen depletion and thrombocytopenia during haemostatic resuscitation after blunt trauma. Conversely, in penetrating trauma, rapid surgical haemorrhage control remains the cornerstone of management.

The present study has several strengths. It is based on a large, registry-based cohort from a well-defined trauma system with high data completeness. The integration of registry data with detailed physiological and laboratory measurements allowed for a comprehensive comparison of early responses to injury. Furthermore, the inclusion of a predefined early transfusion subgroup enabled more detailed analysis of patients with severe haemorrhage.

Several limitations should be acknowledged. The single-centre design may limit generalisability to other settings, particularly regions with different injury patterns, such as a higher prevalence of firearm-related penetrating trauma. Patients declared dead on arrival were excluded because no in-hospital physiological or laboratory data were available. Furthermore, inclusion of these patients could complicate comparisons between trauma systems, as transport to hospital versus declaration of death at the scene is influenced by local prehospital practices. Minor penetrating injuries not requiring trauma system management were also not represented, limiting generalisability to the full spectrum of penetrating trauma. Although we adjusted for key confounders including age, sex, injury severity, and comorbidity, residual confounding cannot be excluded. Long-term functional outcomes beyond hospital discharge were not assessed. Finally, no formal missing data analysis was performed; all analyses relied on available cases. However, data completeness was high owing to prospectively collected data and systematic registry review, and missing data are not expected to have materially influenced the results.

## Conclusion

Within this Scandinavian trauma system, blunt and penetrating trauma represent distinct clinical entities with different patterns of injury, physiological response, and resource utilisation. Penetrating trauma was characterised by early haemodynamic instability and a greater need for emergency surgery, whereas blunt trauma was associated with early haemostatic alterations, higher rates of traumatic brain injury, and worse functional outcomes. Despite these differences, no significant difference in adjusted 30-day mortality was observed between groups. Recognition of these differences is important for early assessment and prioritisation in trauma management.

## Data Availability

The datasets used and/or analysed during the current study are available from the authors on reasonable request and in compliance with the General data protection regulation and Swedish legislation.

## Abbreviations

ASA: American Society of Anesthesiologists
CI: Confidence Interval
CT: Computed Tomography
GCS: Glasgow Coma Scale
GOS: Glasgow Outcome Scale
INR: International Normalised Ratio
IQR: Interquartile Range
ISS: Injury Severity Score
OR: Odds Ratio
PRBC: Packed Red Blood Cells
SD: Standard Deviation
STROBE: Strengthening the Reporting of Observational Studies in Epidemiology
TBI: Traumatic Brain Injury
